# A Low-Cost Foot-Placed UWB and IMU Fusion-Based Indoor Pedestrian Tracking System for IoT Applications

**DOI:** 10.3390/s22218160

**Published:** 2022-10-25

**Authors:** Khawar Naheem, Mun Sang Kim

**Affiliations:** Center for Healthcare Robotics, School of Integrated Technology, Gwangju Institute of Science and Technology, Gwangju 61005, Korea

**Keywords:** data fusion, NLOS, indoor pedestrian positioning, IoT, IMU, PDR, sensor network, UWB, wearables

## Abstract

Among existing wireless and wearable indoor pedestrian tracking solutions, the ultra-wideband (UWB) and inertial measurement unit (IMU) sensors are the popular options due to their accurate and globally referenced positioning, and low-cost and compact size, respectively. However, the UWB position accuracy is compromised by the indoor non-line of sight (NLOS) and the IMU estimation suffers from orientation drift as well as requiring position initialization. To overcome these limitations, this paper proposes a low-cost foot-placed UWB and IMU fusion-based indoor pedestrian tracking system. Our data fusion model is an improved loosely coupled Kalman filter with the inclusion of valid UWB observation detection. In this manner, the proposed system not only adjusts the consumer-grade IMU’s accumulated drift but also filters out any NLOS instances in the UWB observation. We validated the performance of the proposed system with two experimental scenarios in a complex indoor environment. The root mean square (RMS) positioning accuracy of our data fusion model is enhanced by 60%, 53%, and 27% compared to that of the IMU-based pedestrian dead reckoning, raw UWB position, and conventional fusion model, respectively, in the single-lap NLOS scenario, and by 70%, 34%, and 12%, respectively, in the multi-lap LOS+NLOS scenario.

## 1. Introduction

With the emergence of the Internet of Things (IoT), indoor location-based services (ILBSs) to assist daily life tasks (such as smart healthcare, real-time mobility monitoring, etc.) are becoming a trending topic [1,2,3]. The key issue for ILBSs is to accurately locate the pedestrians (patients) indoors [4]. For precise pedestrian tracking, although, non-wearable vision-based solutions are available [5,6,7]; these are expensive, passive, and short-range systems [6]. Moreover, these solutions are unsafe for user privacy and sensitive to indoor light illumination and occlusion [7]. Therefore, there is a demand for affordable, compact-size, accurate, globally referenced, active, and wide-range wearable indoor pedestrian tracking solutions. To meet the target application’s demands, the candidate solutions can be distributed into the globally (e.g., WiFi, Bluetooth (BLE), UWB, etc.) and locally referenced (e.g., pedestrian dead reckoning (PDR)) indoor pedestrian tracking systems [8,9]. The positioning accuracy of the WiFi and BLE is at the meter-level, and so cannot be rated as a high-precision indoor pedestrian tracking system. On the other hand, the UWB-based indoor pedestrian tracking system provides a centimeter-level positioning accuracy [10].

The UWB positioning system is generally considered a high-precision tracking solution. Nevertheless, it requires a clear line of sight (LOS) to guarantee the centimeter-level error [11,12]. For that reason, the UWB positioning accuracy drops significantly in a complex indoor environment where the UWB signal is blocked by obstacles such as furniture and building infrastructure (walls and pillars) [13]. To address the indoor NLOS problem using the UWB positioning system, numerous research schemes have been conducted. Barral et al. [14] present the machine learning- (ML) based NLOS recognition and mitigation approach for low-cost UWB devices. However, their approach requires a custom-designed firmware to access the UWB signal’s features, such as CIR (channel impulse response) and RSS (received signal strength), followed by the storage of a big dataset to train the ML model, which makes this approach impracticable for many real-time applications due to its heavy computation load. Jiménez et al. [15] tackle the UWB indoor NLOS issue by increasing the number of UWB beacons (anchors) up to 12. This scheme has the advantage of realizing an accurate positioning but using too many anchors increases the system cost and requires an optimal survey to set up anchors in the frequent NLOS region. Elsanhoury et al. [16] suggest a sensor fusion to trade off the UWB indoor NLOS instances with other systems’ observations, such as PDR. The sensor fusion seems more practical for real-time indoor pedestrian tracking as it does not require complex and deep processing to distinguish and mitigate the NLOS instances in the UWB position measurements.

The PDR positioning system is based on the IMU sensor (a combination of the accelerometer and gyroscope) to estimate the pedestrian’s position relative to the starting point [9]. The IMU can track pedestrians at faster sample rates with a low-cost and compact size. At the same time, the IMU is robust against external environmental factors such as indoor NLOS and magnetic field interference. On the other hand, the IMU results in high-precision position tracking only for the short-term and deteriorates the position accuracy in longer distances, especially multi-lap trajectories, due to the accumulation of the gyroscope’s drift [17]. Furthermore, the IMU tracks the pedestrian position relative to the starting position, not in the global frame. The magnetometer is usually integrated with the IMU to correct the orientation drift accumulation [18] and improve the tracking accuracy in the global frame, i.e., earth north [19]. The downside of magnetometers is their strong susceptibility to indoor magnetic field interference caused by electronic appliances [20]. In addition, an initial calibration is needed to match the heading angle with the building reference frame [19]. The UWB, due to its insensitivity to magnetic field interferences, could be a better substitute for magnetometers for fusion with the IMU. Corrales et al. [21] and Hol et al. [22] have successfully demonstrated the UWB+IMU data fusion’s ability to address the IMU drift issue in regard to accurate tracking of the human subject in the building/global reference frame.

According to [20], shoes are the preferable wearable mount for human tracking use cases due to: (i) the foot is the primary limb for active detection of an individual’s walking behavior and position change; (ii) the integration of technology on shoes is relatively non-obtrusive and non-stigmatizing, which increase the pedestrian’s acceptance and long-term adherence. Therefore, for the effective utilization of the proposed system, we placed our low-cost UWB+IMU wearable module on the pedestrian’s right shoe. Focusing on the fusion of a foot-placed UWB+IMU wearable, few research studies have been reported to improve the pedestrian’s indoor position accuracy. Fischer et al. [23] propose a precise pedestrian tracking solution using a foot-placed IMU sensor. Still, their outcome is applicable only for a quality-grade expensive IMU sensor which usually generates a stable state estimation with negligible drift. Zihajehzadeh et al. [24,25] design the pedestrian’s lower body motion tracking system using UWB+IMU data fusion. However, their UWB+IMU fusion depends upon the multi-estimator and a magnetometer [25] and does not demonstrate the performance under the NLOS. Zhang, H. et al. [26] propose an EKF-based cost-effective foot-placed UWB+IMU fusion. Yet, their results do not show the effectiveness against the UWB NLOS and long-term IMU drift. Zhang, Y. et al. [27] suggest a robust solution against the UWB NLOS but using the multi-estimator, such as particle filter (PF) and EKF, and investigated the suggested solution only for the short-term IMU drift in a single lap. Nilsson et al. [28] present a dual-foot cooperative fusion approach. Their method corrects the long-term IMU drift using the UWB inter-ranges of the moving agents but in the relative frame. Xu et al. [29,30] describe the novel fusion approaches to address the NLOS. However, they mounted the UWB tag on the upper back and the IMU on a foot; moreover, their approaches depend upon the two estimators and a magnetometer. In summary, most of the existing research has shown the dependency on more than one estimator and a magnetometer when solving the UWB NLOS and IMU drift problems. Furthermore, these studies are limited to either revealing the robustness against the IMU drift or UWB NLOS but not both issues in the same study.

Unlike previous research works, our proposed work addresses the UWB NLOS and IMU drift issues of a low-cost foot-placed UWB and IMU module using a single estimator without the need for a magnetometer. The main contributions of this paper are summarized as follows:

- We propose a low-cost foot-placed UWB and IMU fusion-based indoor pedestrian tracking system to overcome the practical limitations of UWB and IMU wearable sensors. Our data fusion model processes the valid UWB observation by inspecting the residual error to exclude any NLOS instances. As a result, it tackles the UWB’s indoor NLOS and IMU’s accumulated drift issues; it provides a simple but effective indoor pedestrian tracking solution for IoT applications.
- The system hardware is built using off-the-shelf devices. We assembled a prototype of a foot-placed UWB and IMU module to shape a compact-sized battery-powered wearable IoT device, in addition to reducing its cost by up to USD 40, and to incorporate the open-platform software for facilitating the flexible data handling needs of an IoT use case with no additional expense.
- The performance of our system is validated using a Hokuyo Lidar in comparison with an IMU-based PDR, raw UWB position, and conventional fusion model. We conducted two experimental scenarios, a single-lap NLOS and a multi-lap LOS+NLOS, in a complex indoor environment to demonstrate the robustness of our solution against the UWB indoor NLOS and IMU long-term drift.

The remainder of this paper is organized as follows. Section 2 describes the materials and methods required for our proposed system implementation including hardware, the operation principle of the UWB positioning, and the detailed structure of the proposed UWB and IMU fusion. Section 3 and Section 4 present the experiment description and experiment results, respectively, of the proposed data fusion. The discussion and future work appear in Section 5. The conclusion is summarized in Section 6.

## 2. Materials and Methods

**Figure 1:**
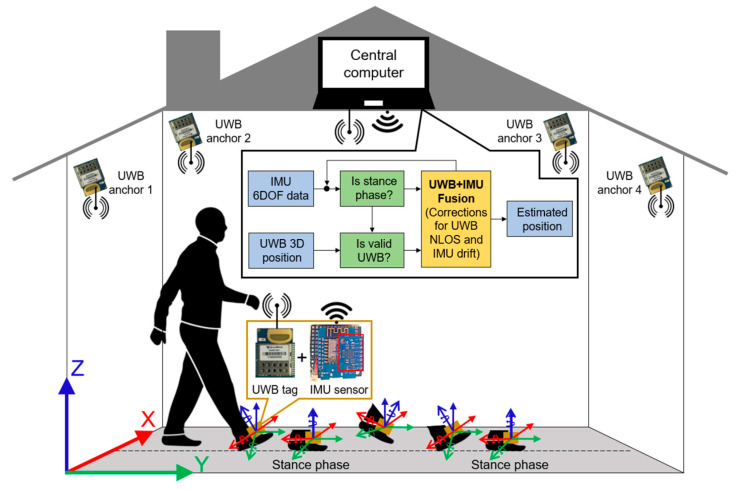
Block diagram of a low-cost foot-placed UWB and IMU fusion-based indoor pedestrian system.

In this section, we explain our proposed system (Figure 1) by first presenting the system hardware and data acquisition in terms of characterizing the UWB sensor, showing the design of a low-cost foot-placed module, and describing the working principle of the UWB positioning system. In the end, we illustrate the proposed UWB+IMU fusion structure in detail.

### 2.1. System Hardware and Data Acquisition

#### 2.1.1. UWB Sensor Characterization

We utilized a UWB sensor network called MDEK1001 (DecaWave, Ireland) [31] to track the foot-placed UWB tag’s position. The MDEK1001 development kit consists of twelve DWM1001DEV and costs about USD 199 [32], which is relatively low. Moreover, each DWM1001DEV is based on the DWM1001C chip and can be assigned to multiple operating modes such as anchor, tag, and data receiver called listener and bridge. We adopted the bridge option as it suits well the IoT application. The anchor and bridge are static devices while the tag is a movable device. The DWM1001DEV can be easily configured via an Android smartphone over BLE to specify each device’s operation mode and to set up the UWB infrastructure. The currently deployed UWB infrastructure comprises four anchors and one gateway which are mounted on the ceiling and wall, respectively. Moreover, the coordinates of the four anchors are manually measured using a precise instrument, i.e., the BOSCH GLM 150C laser range finder. The four anchors perform the TWR measurements with a tag and the 3D position is calculated at the tag, similar to the concept illustrated in Section 2.1.2. The data acquisition from the UWB infrastructure is achieved by connecting a bridge with the Raspberry PI 3B+ header, which is collectively regarded as a gateway. The gateway receives the tag position over the UWB channel and publishes it over the WLAN (wireless local area network) as an MQTT (message queuing telemetry transport) message for the external use case. The MQTT message can be subscribed to the cloud anywhere with a specified message topic. The gateway topology also supports expanding the UWB coverage and forming a multi-room and multi-agent indoor tracking system [31]. In this work, all UWB sensors, i.e., six DWM1001DEV, are configured with the UWB parameters of channel 5, data rate 6.81 Mbps, PRF (pulse repetition frequency) 64 MHz, preamble length 128, and preamble code 9.

**Figure 2:**
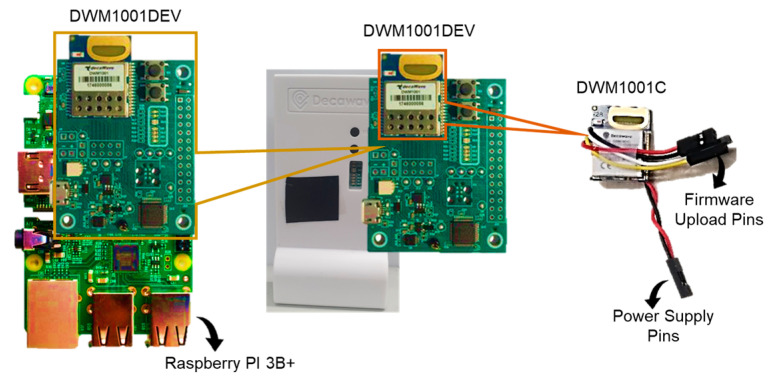
Characterization of UWB sensors (left side = bridge; center = anchor; right side = customized tag).

As the tag is supposed to be foot-placed, it is preferable to mount minimalistic hardware rather than a standard DWM1001DEV (dimensions = 62.0 mm × 43.0 mm). For this sake, we reclaimed a DWM1001C chip (dimensions = 26.2 mm × 19.1 mm) from the sixth DWM1001DEV and soldered the necessary pins, i.e., the power supply and firmware upload, to operate the customized DWM1001C like a standard tag. Figure 2 characterizes the UWB sensor hardware as an anchor, bridge, and customized tag.

#### 2.1.2. Working Principle of UWB Positioning System

**Figure 3:**
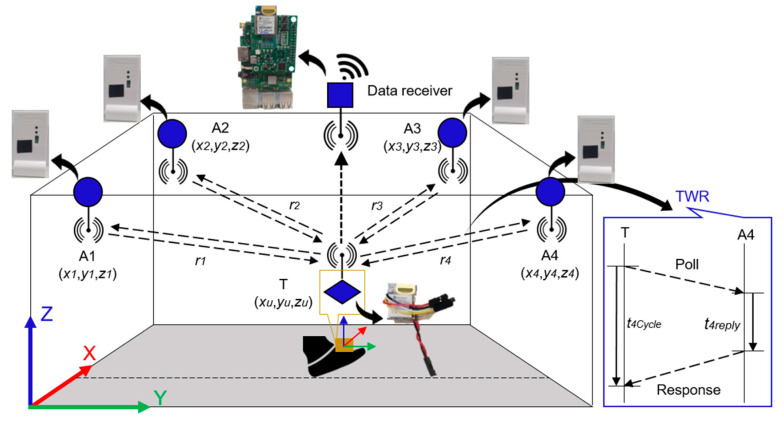
Infrastructure layout and concept of UWB positioning system.

The UWB positioning system works similarly to any other wireless sensor network (WSN) [16] in which some sensors should be deployed on known coordinates and some kept floating on unknown coordinates. The sensors with known coordinates are called anchors ($A_{i}$) while the sensors with unknown coordinates are called tags (T). To locate a tag in two dimensions (2D), at least three anchors are required, and locating a tag in three-dimension (3D) space requires at least four anchors. Figure 3 briefly introduces the concept of a UWB positioning system to locate a tag in 3D using four anchors with known position coordinates.

Generally, the position of a tag in a UWB positioning system can be computed in three steps. Firstly, the anchors’ coordinates can be measured manually using a precise instrument such as a handheld laser range finder to decrease human error. Secondly, the ranges can be measured among anchors and tags using the two-way ranging (TWR) method [16]. Finally, an algebraic trilateration technique [16] can be applied to calculate the position of a tag as presented in (1) and (2).

(1) $$\left(x_{U}^{\prime },y_{U}^{\prime },z_{U}^{\prime }\right)=\overset{4}{\underset{i=1}{\sum }}{\left[\sqrt{{\left(x_{i}-x_{U}\right)}^{2}+{\left(y_{i}-y_{U}\right)}^{2}+{\left(z_{i}-z_{U}\right)}^{2}}-r_{i}\right]}^{2}$$

(2) $$r_{i}=c\frac{\left(t_{iCycle}-t_{iReply}\right)}{2}$$

where i is the number of anchors, i.e., i = 1, 2, 3, 4, $\left(x_{i},y_{i},z_{i}\right)$ is the known coordinates of each anchor, $\left(x_{U},y_{U},z_{U}\right)$, and $\left(x_{U}^{\prime },y_{U}^{\prime },z_{U}^{\prime }\right)$ are the unknown tag positions before and after error minimization of an algebraic trilateration technique [16], respectively. $r_{i}$ is the TWR measurement between each anchor and a tag. $t_{iCycle}\;$ is the time period on a tag from sending the poll message to receiving the response message, $t_{iReply}\;$ is the time period on an anchor from receiving the poll message to sending the response message, and c (= 3 × 10^8^ m/s) is the speed of light. In this work, for a quick and easy integration of off-the-shelf hardware into our system, we relied on the MDEK1001 positioning and networking stack (PANS) library [31] for the UWB positioning.

#### 2.1.3. Wearable IoT Device: A Low-Cost Foot-Placed UWB and IMU Module

**Figure 4:**
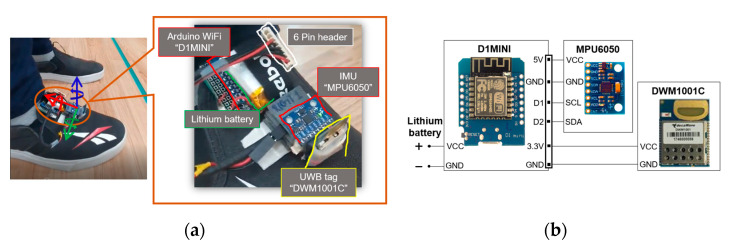
Wearable IoT device. (**a**) Placement of UWB and IMU module at right shoe; (**b**) circuit diagram of custom board.

To qualify as an IoT device, our designed prototype is compact-sized, battery-powered, and capable of communicating both with fellow devices and a central computer over a wireless interface such as UWBs and WiFi, respectively. Our module, as shown in Figure 4a, is placed on the right shoe of the pedestrian. It includes a DWM1001C as the UWB tag, a D1MINI (WEMOS, China) as the WiFi transceiver, an MPU6050 (InvenSense, USA) as the IMU, and a lithium battery 650 mAh as the power source. The lithium battery is directly connected to D1MINI which further powers the DWM1001C at 3.3 V. In addition, both the DWM1001C and D1MINI can also process information onboard. The DWM1001C runs the default PANS library to measure the four anchors’ ranges, and to compute and then transmit the 3D position over the UWB channel for the external use case. Meanwhile, the D1MINI executes the Arduino firmware for the acquisition of the IMU 6 degree-of-freedom (DOF) data via I2C communication, initial calibration of the IMU to remove offsets caused by the manufacturing, and transmission of the calibrated 6DOF data stream over WiFi UDP (user datagram protocol) to the outer application. Figure 4b highlights the circuit diagram of the custom board to accommodate the above-mentioned tasks. The sampling rates of the UWB 3D position and the IMU 6DOF data measurements are set to 10 Hz and 100 Hz, respectively, and the lithium battery can last up to 5.30 h at these sampling rates. The IMU has a sensitivity of 8 g for the accelerometer and 2000°/s for the gyroscope. Table 1 illustrates the specifications of our module.

### 2.2. Proposed UWB and IMU Fusion Structure

**Figure 5:**
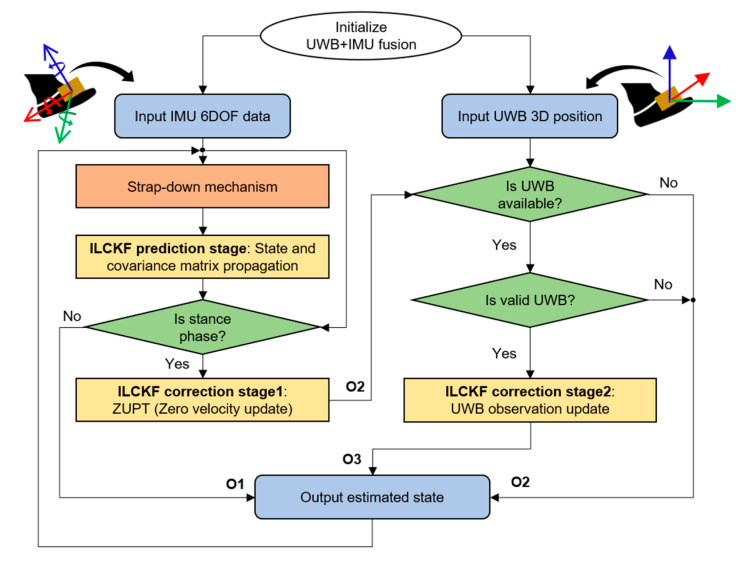
Structure of proposed UWB+IMU fusion.

Theoretically, the correctly initialized IMU can produce accurate state estimates after zero velocity update (ZUPT) [23]. However, it is not applicable for the consumer-grade low-cost IMU in which the errors accumulate swiftly due to the mathematical integration and small measurement biases. The globally referenced sensor such as a magnetometer is normally fused to improve the position accuracy. Still, the magnetometer is strongly influenced by indoor magnetic field interference caused by electronic appliances and needs extra calibration to match the building reference frame. In this paper, we fuse the UWB position observation with the ZUPT-assisted IMU algorithm, which is an improved loosely coupled Kalman filter (ILCKF), as we directly read the UWB position from the MDEK1001 PANS library. Figure 5 portrays the insight into the proposed UWB and IMU fusion structure. For a clear understanding of our fusion structure, we added three flow points, O1, O2, and O3, to indicate the outputs from the strap-down mechanism, ZUPT-assisted IMU, and ILCKF, respectively.

**Figure 6:**
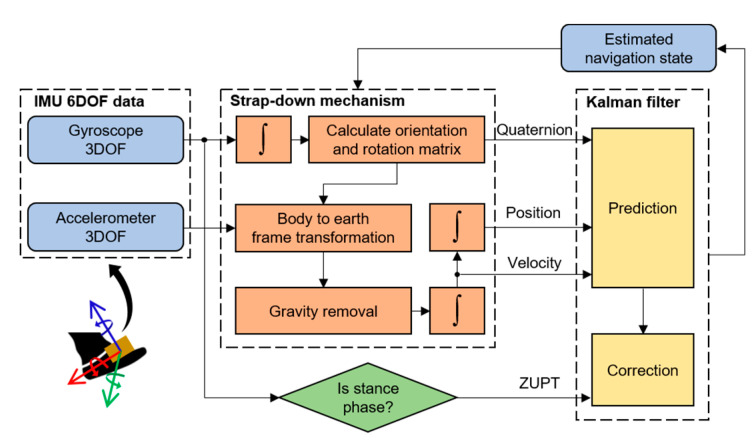
A typical ZUPT-assisted IMU navigation system.

The ZUPT-assisted IMU is a fundamental approach for PDR when the IMU sensor is placed on a foot [23,34]. It mainly consists of a Kalman filter with the strap-down mechanism-assisted state propagation and ZUPT-assisted state correction stages. Figure 6 details the block diagram of a typical ZUPT-assisted IMU navigation system.

#### 2.2.1. Strap-Down Mechanism

We used a set of equations that estimates the inertial navigation state of the IMU in three parts. First, it integrates the gyroscope’s turn rates to calculate the orientation and rotation matrix. Second, it transforms the accelerometer’s forces from the body to the earth’s navigation frame and removes the earth’s gravity. At last, it integrates the transformed accelerometer’s forces to calculate the velocity followed by the position. The basic equation utilizing the IMU 6DOF data to compute the propagated navigation state [34] is as follows

(3) $$\left[\begin{array}{c} p_{k}\\ v_{k}\\ q_{k} \end{array}\right]=\left[\begin{array}{c} p_{k-1}+v_{k-1}dt\\ v_{k-1}+(q_{k-1}a_{k}q_{k-1}^{*}-g)dt\\ \frac{1}{2}\Omega \left(w_{k}dt\right)q_{k-1} \end{array}\right]$$

where k is the time iterator, dt is the time differential, $g={\left[\begin{array}{ccc} 0 & 0 & 9.81 \end{array}\right]}^{T}$ is the gravity, $a_{k}\in {\mathbb{R}}^{3}$ are the accelerometer’s forces in the body frame, $w_{k}\in {\mathbb{R}}^{3}$ are the gyroscope’s turn rates, $p_{k}\in {\mathbb{R}}^{3}$ is the pedestrian’s position, $v_{k}\in {\mathbb{R}}^{3}$ is the pedestrian’s velocity, and $q_{k}\in {\mathbb{R}}^{4}$ is the quaternion describing the system orientation. The triple product $q_{k-1}a_{k}q_{k-1}^{*}\;$ denotes the rotation of $a_{k}$ by $\;q_{k}$, and $\Omega \left(w_{k}\right)$ is the skew-symmetric matrix of the quaternion form of angular velocity.

#### 2.2.2. ILCKF Prediction Stage

The Equations (4)–(6) describe the ILCKF prediction stage.

(4) $$X_{k}=\begin{array}{ccc} {}[p_{k} & v_{k} & q_{k} \end{array}{]}^{T}$$

(5) $$P_{k}=F_{k}P_{k-1}F_{k}^{T}+Q_{k}$$

(6) $$\left.\begin{array}{c} \;Q_{k}=\;\left[\begin{array}{ccc} 0_{3\times 3} & I_{3\times 3}dt & 0_{3\times 4}\\ 0_{3\times 3} & I_{3\times 3}\sigma_{a}^{2}dt & 0_{3\times 4}\\ 0_{4\times 3} & 0_{4\times 3} & \sigma_{w}^{2}s_{qk}s_{qk}^{T}dt \end{array}\right]\\ s_{qt}=\;\frac{1}{2}\left[\begin{array}{c} \begin{array}{ccc} -q_{yk} & -q_{zk} & \;-q_{wk}\\ \;q_{xk} & -q_{wk} & \;q_{zk} \end{array}\\ \begin{array}{ccc} \;q_{wk} & q_{xk} & -q_{yk}\\ -q_{zk\;} & \;q_{yk\;} & q_{xk} \end{array} \end{array}\right] \end{array}\right\}$$

where $X_{k\;}\in {\mathbb{R}}^{10}$ is the state propagation, $P_{k}$ is the propagated covariance matrix, $F_{k}$ is the state transition matrix which can be deduced from Equation (3), and $Q_{k}$ is the process noise covariance matrix.${\;\sigma }_{a}\;$ and $\sigma_{w}$ are the standard deviations of accelerometer and gyroscope noises, respectively.$\;s_{qk}s_{qk}^{T}$ represents the quaternion dynamics. $0_{m\times n}$ and $I_{m\times n}$ (m  is the number of rows and n is the number of columns) are the zero and identity matrices, respectively.

#### 2.2.3. ILCKF Correction Stage1: ZUPT

**Figure 7:**
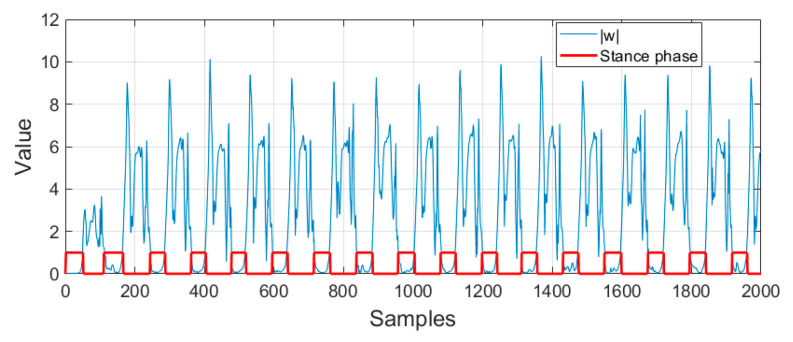
Result of implemented stance phase detection.

For the IMU’s orientation drift compensation, the ZUPT is applied periodically at every stance phase detection [23] as follows

(7) $$\mathrm{stance}\;\mathrm{phase}=\left\{\begin{array}{c} 1;\;when\;\left|w\right|\leq \psi \;\\ 0;\;when\;\left|w\right|>\psi \; \end{array}\right.$$

where $\left|w\right|$ is the magnitude of gyroscope 3DOF values and ψ is the zero velocity detection threshold. Figure 7 describes the result of stance phase detection in a red color square waveform. Now, the ILCKF correction stage for the ZUPT can be expressed as

(8) $${\hat{X}}_{k}=X_{k}+K_{k}(0_{1\times 3}-v_{k})$$

(9) $${\hat{P}}_{k}=(I_{10\times 10}-K_{k}H)P_{k}$$

(10) $$\left.\begin{array}{c} \;K_{k}=P_{k}H^{T}{\left(HP_{k}H^{T}+R\right)}^{-1}\\ \;H=\left[\begin{array}{ccc} 0_{3\times 3} & I_{3\times 3} & 0_{3\times 4} \end{array}\right])\\ \;R=I_{3\times 3}\sigma_{v}^{2}\; \end{array}\right\}$$

where ${\hat{X}}_{k}$ is the corrected navigation state, ${\hat{P}}_{k}\;$ is the corrected covariance matrix, and $K_{k}$ is the Kalman gain.$\;0_{1\times 3}\;$ is the ZUPT observation value, H and R are the ZUPT observation matrix and observation noise covariance matrix, respectively, and $\sigma_{v}\;$ is the standard deviation of zero velocity noise.

#### 2.2.4. ILCKF Correction Stage2: UWB Observation Update

**Figure 8:**
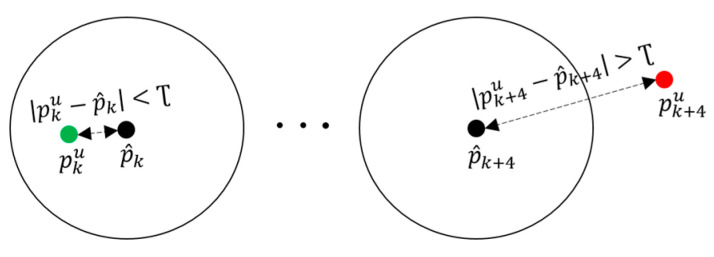
Block diagram of valid UWB observation detection having valid UWB position at left side and invalid UWB position at right side. (Black dot = IMU estimated position; green dot = UWB position in LOS; red dot = UWB position in NLOS.)

It is understood that the UWB positioning accuracy degrades in NLOS and upgrades in LOS. Thus, to ensure the LOS condition, we checked the validity of every UWB observation by making use of the short-term accurate positioning characteristics of the IMU (Section 2.2.3). The valid UWB position detection is expressed as follows

(11) $$\mathrm{valid}\;\mathrm{UWB}=\left\{\begin{array}{c} 1;\;when\;\left|p_{k}^{u}-{\hat{p}}_{k}\right|\leq Ʈ\;\\ 0;\;when\;\left|p_{k}^{u}-{\hat{p}}_{k}\right|>Ʈ\; \end{array}\right.$$

where $p_{k}^{u}\in {\mathbb{R}}^{3}$ is the UWB position observation, ${\hat{p}}_{k}\in {\mathbb{R}}^{3}$ is the ZUPT corrected position, $\left|p_{k}^{u}-{\hat{p}}_{k}\right|$ is the absolute residual error, and Ʈ  is the threshold value which is also regarded as a radial distance. Figure 8 presents the block diagram of valid UWB observation detection.

After the success of the UWB position validity check, the current UWB position observation is processed in the second ILCKF correction stage as stated in Equations (12)–(14).

(12) $${\hat{X}}_{k}^{u}={\hat{X}}_{k}+K_{k}^{u}\left(p_{k}^{u}-{\hat{p}}_{k}\right)$$

(13) $${\hat{P}}_{k}^{u}=(I_{10\times 10}-K_{k}^{u}H_{k}^{u}){\hat{P}}_{k}$$

(14) $$\left.\begin{array}{c} \;K_{k}^{u}={\hat{P}}_{k}H^{uT}{\left(H^{u}{\hat{P}}_{k}H^{uT}+R^{u}\right)}^{-1}\\ \;H^{u}=\left[\begin{array}{ccc} I_{3\times 3} & 0_{3\times 3} & 0_{3\times 4} \end{array}\right])\\ R^{u}=I_{3\times 3}\sigma_{u}^{2} \end{array}\right\}$$

where $\;{\hat{X}}_{k}^{u}$ is the UWB corrected navigation state, ${\hat{P}}_{k}^{u}\;$ is the UWB corrected covariance matrix, and $K_{k}^{u}$ is the UWB Kalman gain. $H^{u}$ and $R^{u}$ are the UWB observation matrix and observation noise covariance matrix, respectively, and $\sigma_{u}\;$ is the standard deviation of the UWB position noise. In all equations, the non-italic symbols represent the constant values and matrices. 

The presence of valid UWB position detection improves the reliability of our fusion model by enabling pedestrian tracking in an NLOS-prone indoor environment. Moreover, the dependency on a simple position difference (i.e., an absolute residual error) transforms our system into a practical solution for IoT applications.

## 3. Experiment Description

This section describes the experimental setup used for the validation of the developed algorithm along with the data processing at the central computer and performance criteria.

### 3.1. Experimental Setup

**Figure 9:**
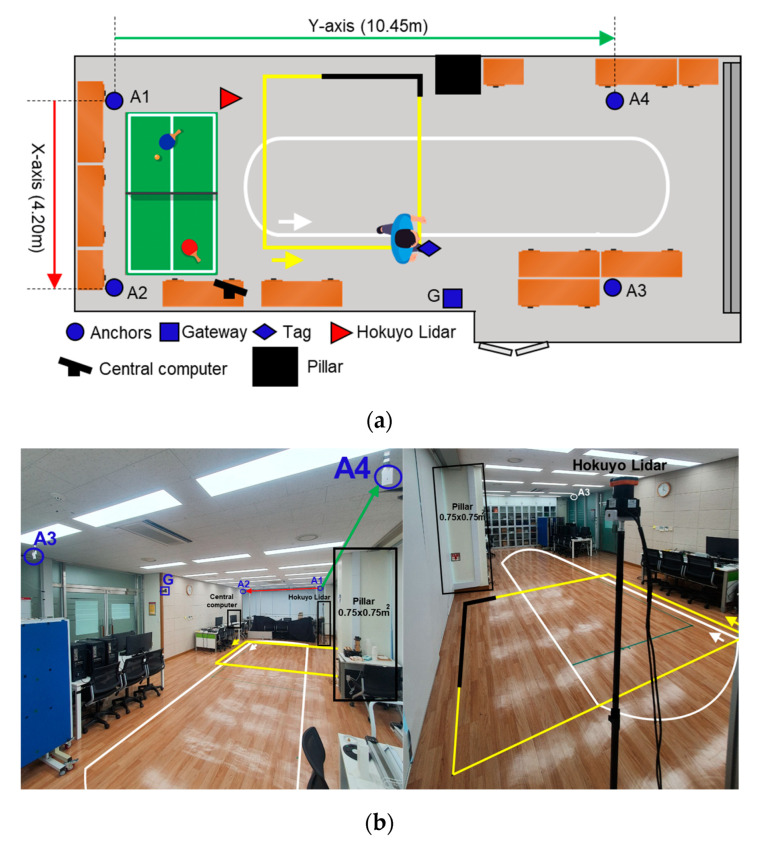
Experiment environment. (**a**) Top view of experimental setup; (**b**) site photos with two views of hardware deployment.

We conducted the pedestrian tracking experiment in Room 513, Dasan Building, Gwangju Institute of Science and Technology (GIST). In our experiment, the pedestrian was a healthy male subject of 34 years of age and 1.78 m tall. Figure 9 details the experimental space which comprised furniture and a pillar, which in general is a complex indoor environment. In order to investigate the qualitative and quantitative performances of the proposed fusion scheme, we deployed a state-of-art laser sensor, a Hokuyo Lidar UTM-30LX (Japan), which has a 0.1 m–30 m range, ±30 mm accuracy from a 0.1 m–10 m range, ±50 mm accuracy from a 10 m–30 m range, a 270° horizontal field of view, and a 25 ms scan speed. The Hokuyo Lidar was mounted on a tripod at the pedestrian’s head height for easy tracking of the pedestrian’s center position.

**Figure 10:**
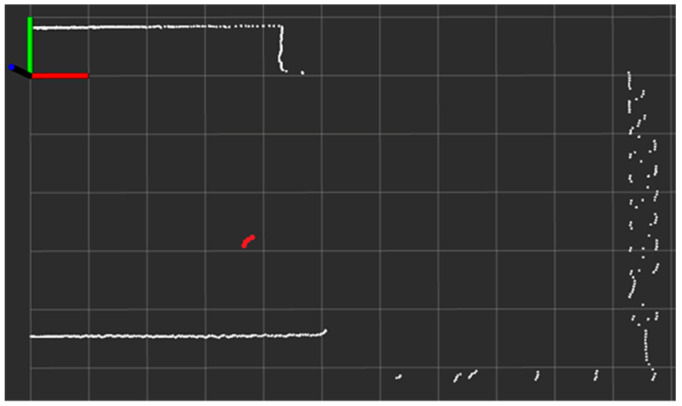
ROS visualization of real-time Hokuyo Lidar’s scan in Hokuyo reference frame of same scene portrayed in Figure 9a (white dots = redundant surrounding boundaries; red dots = pedestrian’s head position; per unit box = 1 m × 1 m).

In Figure 9, the blue circles and the square are the UWB sensors, the red triangle is a Hokuyo Lidar, the large black square is a pillar, and the other icons are furniture. Furthermore, the yellow and white trajectories describe the two reference paths on which the pedestrian walked at normal walking velocity during the experiment. The yellow trajectory indicates the first experimental scenario, named the single-lap NLOS, and the addition of a white trajectory alongside the yellow trajectory formulates the second experimental scenario, called the multi-lap LOS+NLOS. More details about the experimental scenarios are provided in Section 4. Table 2 lists the coordinates needed for our setup configuration. Figure 10 shows the pedestrian’s head segmentation in the Hokuyo frame with red dots and the raw point cloud with white dots. The centroid or center position of the segmented points is the desired ground truth position of the pedestrian.

### 3.2. Data Handling at Central Computer

**Figure 11:**
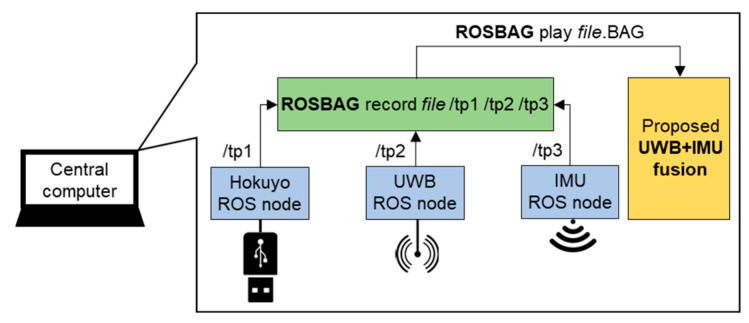
Data recording and post-analysis structure using ROS framework.

The overall data processing required for our experiment setup is portrayed in Figure 11. The ROS (robot operating system) framework records data as a ROSBAG file synchronized at the central computer’s clock, even though the data are streaming from three unsynchronized ROS nodes: 40 Hz for Hokuyo, 10 Hz for UWB, and 100 Hz for IMU. The Hokuyo ROS node uses the standard ROS package to access the Lidar over a USB port and publishes the ROSTOPIC /tp1. The UWB and IMU ROS nodes are based on the custom ROS packages. In detail, the UWB ROS node subscribes to the tag 3D position coordinates as an MQTT message from the gateway and publishes the ROSTOPIC /tp2. In addition, the IMU ROS node receives the 6DOF data stream over WiFi UDP and publishes the ROSTOPIC /tp3. Afterward, the recorded ROSBAG file is played for post-analysis of the proposed UWB+IMU fusion under the two experimental scenarios.

### 3.3. Performance Criteria

We selected four criteria—the 2D trajectory, root mean square error (RMSE), cumulative distribution function (CDF), and box plot—to investigate the pedestrian tracking performance of our fusion model in comparison with the IMU-based PDR [23], raw UWB position [31], and conventional fusion [26] algorithms. The positioning errors are calculated based on the RMSE criterion, but after transforming the ground truth position from the Hokuyo frame to the UWB global frame. It should be noted that to extract the realistic positioning error, we shifted the estimated position by 0.1 m (displacement between pedestrian’s head and foot) towards the ground truth position at every stance phase instance. In a box plot, the inner red line and floating red plus of the blue box specify the median and mean values, while the bottom and top edges of the blue box indicate the positioning errors of 25% and 75%, respectively. The outer black whiskers of the blue box show the minimum and maximum errors.

We used MATLAB R2018A (MathWorks, USA) for the implementation and analyses. During initialization, the noise parameters are tuned as $\sigma_{a}=$0.001 m/s^2^, $\sigma_{w}=$0.01 rad/s, $\sigma_{v}=$0.001 m/s, and ψ= 0.6 rad/s for the ZUPT-assisted IMU propagation and update, and $\sigma_{u}=$ 0.2 m and $Ʈ=3\sigma_{u}\;$ for the UWB position update.

## 4. Experiment Results

This section validates and discusses the performance of the proposed system through experimental results under two scenarios.

### 4.1. Single-Lap NLOS Scenario

In this scenario, we revealed the performance of the proposed system mainly under the UWB NLOS situation. To emulate the severe NLOS phenomena in an indoor environment, we considered a path passing by the building infrastructure such as a pillar of 0.75 m × 0.75 m thickness. As a pedestrian crosses the pillar, the UWB anchors, i.e., A3 and A4, are subjected to the UWB signal blocking which ultimately creates an NLOS phenomenon. Figure 9 displays this scenario with a yellow trajectory where the NLOS region is indicated by an overlapped black trajectory. The pedestrian starts walking near the yellow arrow and walks for one lap in a counterclockwise direction.

**Figure 12:**
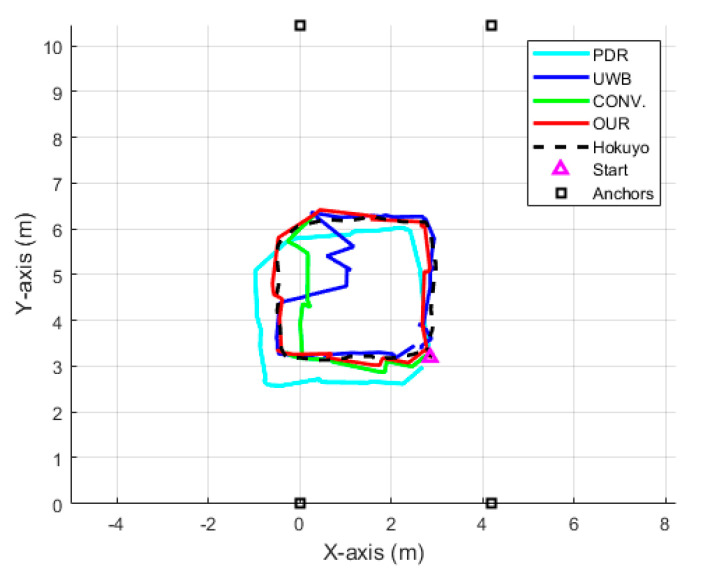
Position trajectory of each algorithm in single-lap NLOS scenario.

**Figure 13:**
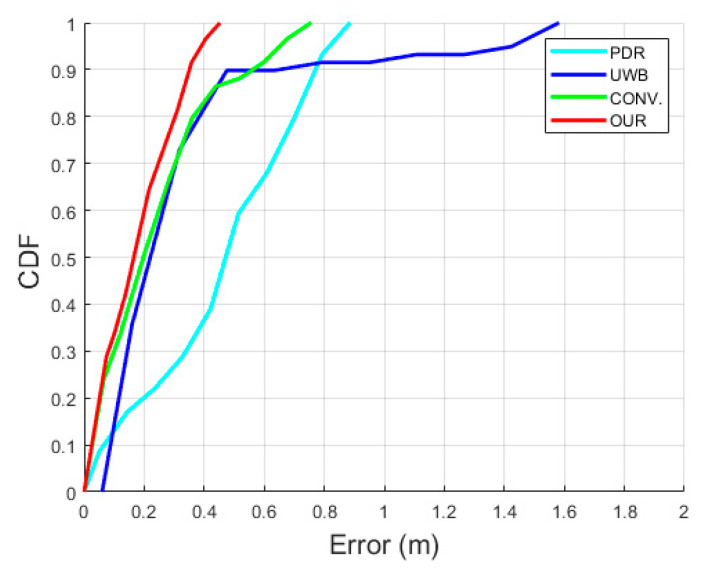
CDF of positioning errors in single-lap NLOS scenario.

**Figure 14:**
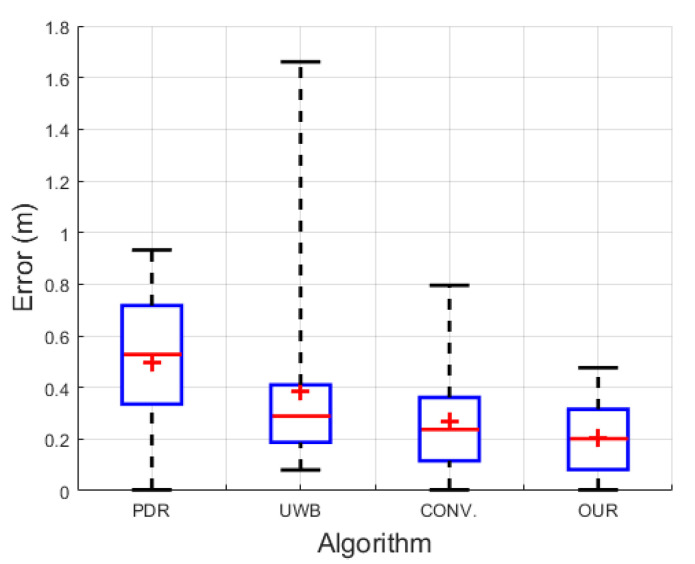
Box plot of positioning errors in single-lap NLOS scenario.

Figure 12 details the visual comparison among the PDR in the cyan line, the UWB positioning system (UWB) in the blue line, the conventional UWB+IMU fusion (CONV.) in the green line, the proposed UWB+IMU fusion (OUR) in the red line, and the Hokuyo reference system in the dotted black line. The PDR trajectory is smooth but has an orientation drift even in a single lap, while the UWB depicts a large error in the NLOS region. On the other hand, the CONV. achieves a slightly improved trajectory compared to the UWB in the NLOS region, but still shows a diverted tail during the NLOS recovery region. Overall, OUR accomplishes a smooth trajectory in both regions, i.e., with and without the NLOS phenomenon. This signifies that the addition of the valid UWB observation detection produces high-precision pedestrian tracking compared to the other three algorithms. The CDF of the positioning errors among all the algorithms is visualized in Figure 13. At the 95th percentile, the UWB, PDR, CONV., and OUR exhibit errors of 1.45 m, 0.80 m, 0.65 m, and 0.40 m, respectively. Figure 14 presents the box plot of the positioning errors for the four algorithms to highlight the mean, median, and extreme points of the positioning errors. It can be deduced from Figure 14 that the UWB shows the worst performance and OUR reflects the best performance. Table 3 states the positioning errors quantitatively as 2D, X-axis, Y-axis, mean, median, and maximum errors. Both Figure 14 and Table 3 depict a similar performance trend where OUR reflects the smallest maximum error compared to the other three algorithms.

In summary, our UWB+IMU fusion accomplishes a more enhanced performance in a complex indoor space than that of the algorithms without the valid UWB observation: 53% and 27% better than raw UWB and conventional fusion models, respectively. It could also be concluded that when the UWB observation is corrupted or the UWB signal is lost in the NLOS region, the proposed fusion model successfully rejects the corrupted UWB observation by trusting the IMU position estimation.

### 4.2. Multi-Lap LOS+NLOS Scenario

This scenario emphasizes the evaluation of the robustness of the proposed system for longer distances, especially against the IMU’s accumulated drift under mixed surrounding situations. For this sake, we emulated a lengthier path just by increasing the same path repetition up to four laps for LOS white trajectory and three laps for NLOS yellow trajectory, as shown in Figure 9. The pedestrian walks non-stop in both trajectories successively and starts walking near the white arrow in a counterclockwise direction. The distance covered in each lap of the LOS white trajectory and NLOS yellow trajectory was about 20 m and 10 m, respectively, so, collectively the pedestrian walked for 110 m, i.e., (4 laps × 20 m) + (3 laps × 10 m).

**Figure 15:**
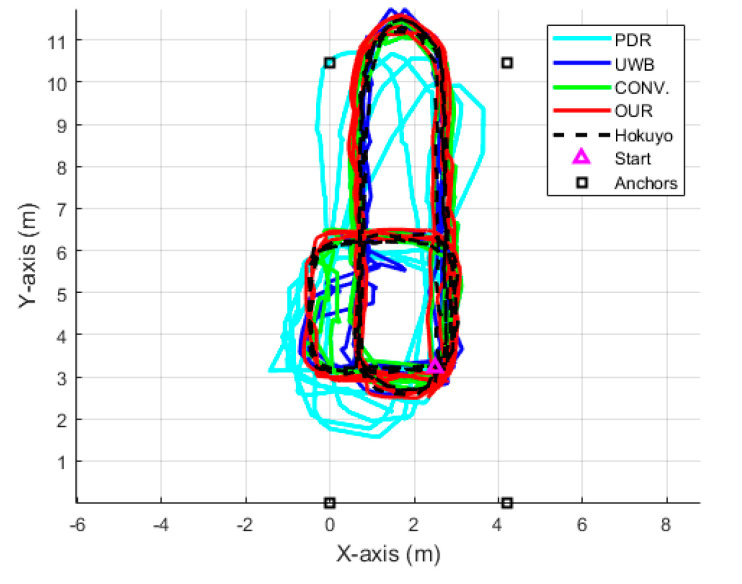
Position trajectory of each algorithm in multi-lap LOS+NLOS scenario.

**Figure 16:**
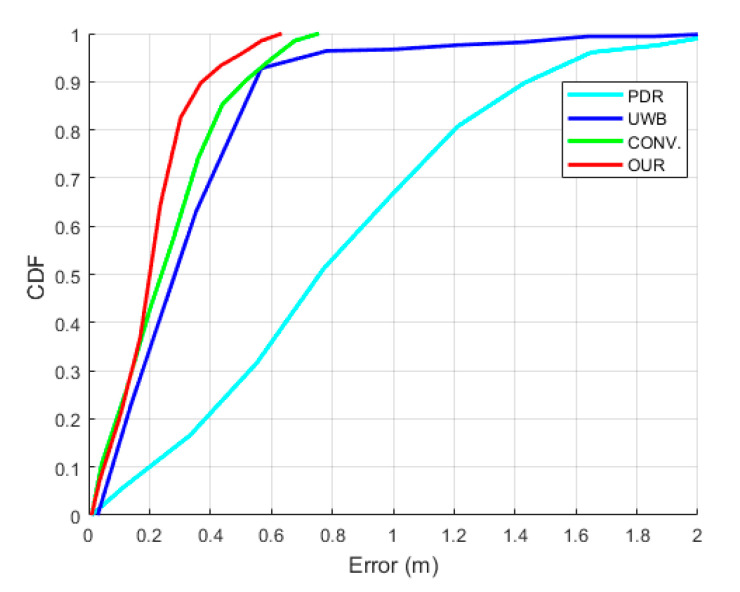
CDF of positioning errors in multi-lap LOS+NLOS scenario.

**Figure 17:**
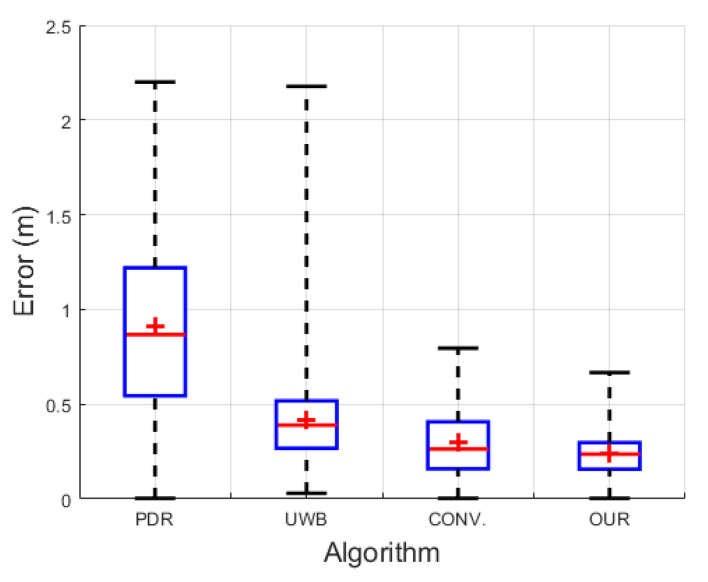
Box plot of positioning errors in multi-lap LOS+NLOS scenario.

Figure 15 presents the comparative 2D position of the trajectories among the various algorithms, similarly to Figure 10. It can be seen that all of the algorithms have similar trajectories except the PDR. Moreover, the UWB position has a consistent and repeatable accuracy throughout the four laps of the LOS trajectory; on the other hand, due to the NLOS situation in the three laps of the NLOS trajectory, the UWB data have considerable noise. The fusion of UWB and IMU sensors significantly enhanced the pedestrian tracking performance in both LOS and NLOS situations, but OUR outperforms the CONV. due to the valid UWB observation detection. Figure 16 shows the comparative CDF of positioning errors among all of the algorithms. Evidently, OUR achieves a 0.45 m positioning error compared to the 1.63 m of the PDR at the 95th percentile. Figure 17 provides a box plot to visualize the mean, median, and extreme values of the positioning errors. Table 4 illustrates the quantitative values in terms of 2D, X-axis, Y-axis, mean, median, and maximum errors. According to Figure 17 and Table 4, the IMU-based PDR is the only algorithm to reveal the larger error values of all performance criteria in contrast with the UWB, CONV., and OUR.

In short, the proposed fusion performs 70%, 34%, and 12% better than PDR, UWB, and CONV., respectively. It can be also observed that the multi-lap pedestrian walking drastically and continuously accumulates the IMU drift; on the other hand, the UWB observation occasionally and discretely experiences the NLOS situation only when the pedestrian passes by the pillar. Hence, it is validated that the inclusion of the valid UWB observation update effectively compensates for the long-term IMU’s drift accumulation as well as UWB indoor NLOS.

## 5. Discussion and Future Work

To address the UWB NLOS and IMU accumulated drift problems, the existing foot-placed solutions for indoor pedestrian tracking usually depend on involving quality-grade expensive hardware [23], ML-based NLOS detection and mitigation [14], and more than one estimator alongside a magnetometer [25,29,30]. This ultimately increases the system’s cost and complexity and makes it susceptible to indoor magnetic field interference. The goal of this work was to develop a low-cost foot-placed UWB+IMU fusion-based indoor pedestrian tracking solution that can overcome the UWB’s indoor NLOS and consumer-grade IMU’s accumulated drift in the long term. The proposed system was evaluated by computing the RMS positioning errors using the pedestrian’s ground truth position obtained from the state-of-the-art Hokuyo Lidar with a ±30 mm accuracy. The pedestrian tracking experiment, comprising of two scenarios, was conducted in a complex indoor environment by walking on the predefined trajectories. Furthermore, the qualitative and quantitative performances were validated in comparison with the three algorithms: IMU-based PDR [23], raw UWB position [31], and conventional fusion model [26]. With the two experimental scenarios, we justified that the IMU estimation helps to select the valid UWB observation which further corrects the long-term IMU accumulated drift. Eventually, the proposed system accomplished the RMS positioning error of 0.29 ± 0.03 m.

**Figure 18:**
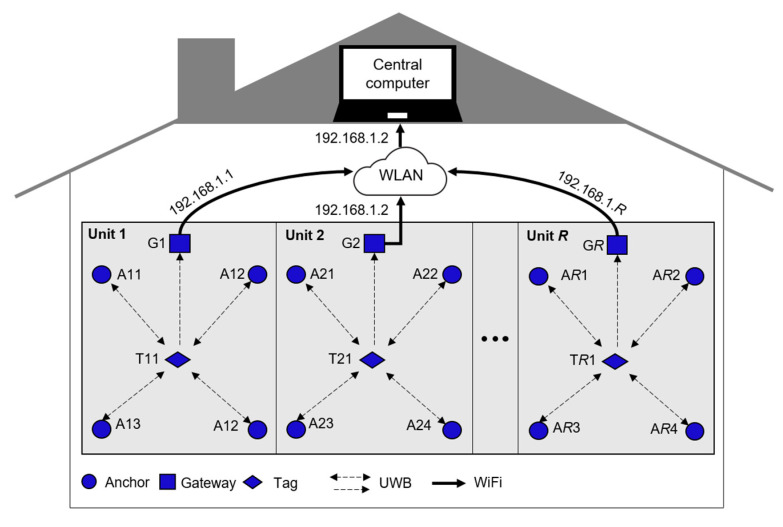
Networking topology to track pedestrians in whole building using UWB sensor network.

Thanks to the off-the-shelf hardware and open-platform software ROS and Arduino, our system can be deployed with a low cost and easy accessibility for accurate indoor pedestrian tracking IoT applications. In addition, the inclusion of the UWB positioning system brings the advantage of actively tracking the pedestrians in a building reference frame at a centimeter-level accuracy. At the same time, the need for special infrastructure is a downside of using the UWB positioning system. So far, the developed system was tested for room-scale single-unit pedestrian tracking in a mixed LOS+NLOS indoor environment. However, in narrow corridors and crowded areas, the pedestrian tracking accuracy using our system might drop due to the long-interval blocking of the UWB signal and the severity of the NLOS situation compared to inside the rooms. In the future, we would like to extend our current work by considering dynamic NLOS situations in more complex and realistic indoor environments as well as the consumer-grade IMU parameters uncertainties. We will optimize the sampling rates of IMU and UWB data acquisition to prolong the battery life by reducing power consumption. Moreover, we want to scale up the coverage area of the UWB sensor network to multiple units and track multiple pedestrians by incorporating the MDEK1001 suggested topology as portrayed in Figure 18. Ultimately, we envision our wearable IoT device to be a part of future smart shoes that can track the patients’ walking patterns and automatically diagnose and prevent gait-related issues in real time.

## 6. Conclusions

In this paper, we proposed a low-cost foot-placed UWB and IMU fusion-based indoor pedestrian tracking system. Our system fused the off-the-shelf UWB and consumer-grade IMU sensors to simultaneously counter their practical limitations such as the UWB’s indoor NLOS and IMU’s accumulated drift problems in a complex indoor environment. It was validated from the two experimental scenarios that the proposed system achieved a superior performance of 53% better than the raw UWB position in the NLOS situation, and 70% better than the IMU-based PDR in the long run. We expect that our outcomes can contribute to the adaption of low-cost wearables in the accurate and intelligent monitoring of pedestrians for indoor IoT applications.

## Figures and Tables

**Table 1:** Specifications of low-cost foot-placed module.

Device Name	Dimensions	Price
DWM1001C	26.2 mm × 19.1 mm × 2.6 mm	USD 18 [32]
MPU6050	21.2 mm × 16.4 mm × 3.3 mm	USD 4 [33]
D1MINI	34.2 mm × 25.6 mm × 7.0 mm	USD 5 [33]
Lithium battery 650mAh	35.0 mm × 20.0 mm × 10.0 mm	USD 3 [33]

**Table 2:** Experimental setup configuration.

Coordinate Value	Anchor Number				Hokuyo Lidar
	A1	A2	A3	A4	
*x*-axis (m)	0	4.20	4.20	0	0
*y*-axis (m)	0	0	10.45	10.45	2.30
*z*-axis (m)	2.51	2.51	2.51	2.51	1.70

**Table 3:** Positioning errors in single-lap NLOS scenario.

Algorithm	2D (m)	*x*-axis (m)	*y*-axis (m)	Mean (m)	Med. (m)	Max. (m)
PDR	0.60	0.53	0.28	0.49	0.52	0.93
UWB	0.52	0.50	0.17	0.38	0.28	1.66
CONV.	0.33	0.29	0.16	0.26	0.23	0.80
OUR	**0.24**	**0.18**	**0.15**	**0.20**	**0.20**	**0.47**

All values have tolerance of ±0.03 m.

**Table 4:** Positioning errors in multi-lap LOS+NLOS scenario.

Algorithm	2D (m)	*x*-axis (m)	*y*-axis (m)	Mean (m)	Med. (m)	Max. (m)
PDR	1.02	0.71	0.73	0.91	0.86	2.20
UWB	0.46	0.35	0.29	0.41	0.38	2.17
CONV.	0.34	0.21	0.26	0.29	0.26	0.80
OUR	**0.29**	**0.18**	**0.24**	**0.24**	**0.24**	**0.66**

All values have tolerance of ±0.03 m.

## Data Availability

The data presented in this study are available from the corresponding author upon reasonable request.
